# A multi-stakeholder approach in optimising patients’ needs in the benefit assessment process of new metastatic breast cancer treatments^☆^

**DOI:** 10.1016/j.breast.2020.04.011

**Published:** 2020-05-10

**Authors:** Fatima Cardoso, Nils Wilking, Renato Bernardini, Laura Biganzoli, Jaime Espin, Kaisa Miikkulainen, Susanne Schuurman, Danielle Spence, Sabine Spitz, Sonia Ujupan, Nicole Zernik, Jenn Gordon

**Affiliations:** a Champalimaud Clinical Centre, Champalimaud Foundation and ABC Global Alliance, Lisbon, Portugal; b Karolinska Institutet, Stockholm, Sweden; c University of Catania Medical School, Catania, Italy; d Hospital of Prato and European Society of Breast Cancer Specialists, Florence, Italy; e Andalusian School of Public Health, Escuela Andaluza de Salud Pública (EASP), Granada, Spain; f CIBER en Epidemiología y Salud Pública / CIBER of Epidemiology and Public Health (CIBERESP), Spain; g Instituto de Investigación Biosanitaria ibs, Granada, Spain; h ICON Plc, Stockholm, Sweden; i ICON Plc, Stockholm, Sweden at the time of article submission; j Breast Cancer Network Australia, Australia; k Europa Donna, Vienna, Austria and European Patients’ Academy on Therapeutic Innovation (EUPATI); l Eli Lilly and Company, Brussels, Belgium; m Europa Donna, Paris, France; n Canadian Breast Cancer Network, Ottawa, ON, Canada

**Keywords:** Metastatic breast cancer, Health technology assessment, Policy, Stakeholders, Treatment benefit, Patient preferences

## Abstract

There is a growing understanding as science evolves that different cancer types require different approaches to treatment evaluation, especially in the metastatic stages. The introduction of new metastatic breast cancer (MBC) treatments may be hindered by several elements, including the availability of relevant evidence related to disease-specific outcomes, the benefit assessment process around the evaluation of the clinical benefit and the patients’ need of new treatments.

The Steering Committee (SC) found that not all issues relevant to MBC patients are consistently considered in the current benefit assessment process of new treatments. Among these are overall survival, time-to-event endpoints (e.g. progression-free survival), patients’ priorities, burden of disease, MBC-specific quality of life, value in delaying chemotherapy, route of administration, side effects and toxicities, treatment adherence and the benefit of real-world evidence. This paper calls on decision makers to (1) Include MBC-specific patient priorities and outcomes in the overall benefit assessments of new MBC treatments; (2) Enhance multi-stakeholder collaboration in order to improve MBC patient outcomes.

## Highlights

• Patient preferences should be considered in MBC treatment decision making.
• The patient perspective should be integrated into treatment guidelines for MBC.
• Patients should be involved in MBC policy decision making.
• Multi-stakeholder collaboration could improve MBC patient outcomes and access to treatments.

## Abbreviations

ABC: Advanced Breast Cancer
ASCO: American Society of Clinical Oncology
BC: Breast Cancer
CADTH: Canadian Agency for Drugs and Technologies in Health
ESMO: European Society for Medical Oncology
ESO: European School of Oncology
EUSOMA: European Society of Breast Cancer Specialists
HRQoL: Health-Related Quality of Life
HTA: Health Technology Assessment
MBC: Metastatic Breast Cancer
MCBS: ESMO Magnitude of Clinical Benefit Scale
MDTs: Multidisciplinary Teams
MTBs: Multidisciplinary Tumour Boards
OS: Overall Survival
pCODR: pan-Canadian Oncology Drug Review
PFS: Progression-Free Survival
PROs: Patient-Reported Outcomes
QoL: Quality of Life
RWE: Real-World Evidence
SMC: Scottish Medicines Consortium

## Introduction

Breast cancer (BC) is the most common cancer in women worldwide, with 2.09 million new cases diagnosed in 2018 [1]. Metastatic breast cancer (MBC) is responsible for the vast majority of the 0.6 million deaths from BC each year globally [1,2]. Although there has been progress in the treatment of MBC over the past decade, it remains an incurable but treatable disease [3].

MBC is associated with a substantial humanistic and economic burden, with a considerable impact on the quality of life of patients and their caregivers, and on healthcare spending and budgets. There are also issues of inequity in patient access to quality care arising from challenges facing healthcare decision makers in their funding decisions regarding new MBC treatments. These challenges include scarce budgetary resources, the trade-off between fast access and valuable evidence to the patients, and the lack of high-quality and mature datasets from clinical trials/practice to inform their decision making [4]. Additionally, current treatment benefit assessments typically do not adequately consider factors that are specific to MBC patients [5].

A Steering Committee (SC) comprising individuals from patient organisations, academia, oncologists and the pharmaceutical industry (see Table 1 for the full overview) was established to examine the challenges in current MBC treatment decision making processes with respect to patients’ needs. This multi-stakeholder group published a call for action to policy makers, healthcare professionals, academia, patient advocates, patients and members of the MBC community to close gaps in the provision of MBC care through collaboration and greater consideration of patients’ needs [6].

**Table 1:** Representatives interviewed from several stakeholder groups.

	Institutions	Area of expertise
Patient organisations	Breast Cancer Network Australia	Policy and advocacy informed by consumer and clinical expert consultations
	The Canadian Breast Cancer Network	Patient experience, health policy, Canadian HTA and advocacy
	Europa Donna Austria^a^	Breast cancer patient advocacy
	Europa Donna France	Breast cancer patient advocacy
	European Patients’ Academy on Therapeutic Innovation ( EUPATI)^a^	Patient advocacy
Clinical and academic institutions	Andalusian School of Public Health (Spain)	HTA, European pharmaceutical policies, public health and health economics
	The University of Catania Medical School (Italy)	Clinical pharmacology; Expertise in regulatory affairs
	The European Society of Breast Cancer Specialists (EUSOMA)	Medical oncology and geriatric oncology
	The Department of Oncology-Pathology at the Karolinska Institutet (Sweden)	Clinical oncologist and access to treatments in Sweden
	Champalimaud Clinical Center/Champalimaud Foundation (Portugal)	Medical Oncology, access to treatments, public policy, cancer research
International organisation	ABC Global Alliance (International, headquarters in Portugal)	Multi-stakeholder organisation fully dedicated to advanced breast cancer patients – access, policy, advocacy, lobbying
Pharmaceutical company	Eli Lilly and Company	Health policy and advocacy

a The same representative for Europa Donna Austria and EUPATI

This consensus paper is based on the SC discussions and non-systematic literature searches of select HTA agency websites and country-specific government, research institutes and patient organisation websites (Appendix). The searches were performed online using the Google search engine between April and May 2018; search terms used were ‘metastatic breast cancer’, ‘metastatic breast cancer AND policy’, ‘metastatic breast cancer AND value’ and ‘metastatic breast cancer AND HTA’ and the searches were global in scope.

### Metastatic breast cancer patients’ needs in the decision making process

Assessing the overall benefit of a new MBC treatment is key to decision makers, such as clinicians, regulatory and payers, and it is also of value to patients. For a new treatment to become available to patients, it must first be assessed for efficacy and safety by medicine regulatory bodies and for relative value and efficacy by HTA or reimbursement bodies. The latter typically take into account the clinical and economic evidence as well as ethical considerations [7]. However, these organisations tend to use different endpoints to assess whether a new treatment should be made available, and there are different approaches to assessing the relative benefit of treatments in HTA appraisals [[8], [9], [10], [11], [12]]. During the discussions, the SC addressed only certain aspects (patients’ needs) regarding the benefit assessment for new MBC treatments and not the entire HTA process.

Overall survival (OS) and progression-free survival (PFS) are key clinical endpoints used in oncology trials, and have different aims of measurement. Regulators are willing to accept PFS as a surrogate endpoint for OS for the purpose of regulatory approval of a new cancer therapy. In contrast, treatment benefit assessment decision makers tend to focus almost exclusively on OS when evaluating the benefit of a new therapy [6]. However, when no OS gain is observed due to immature data, there is usually willingness to consider surrogate endpoints (such as PFS) in the benefit assessments of the clinical evidence. This compromise, where an assessment is undertaken in the absence of OS data, leads to some uncertainty for decision makers while they wait for mature data to become available for an OS analysis.

Given the severe and progressive nature of MBC, it is important in benefit assessments to recognise disease-specific, patient- and disease-relevant outcomes and consider them when making decisions concerning MBC treatments [5]. This multi-stakeholder collaboration calls for alignment on concrete patient-relevant evidence requirements in MBC and a common definition of overall treatment benefit, as outlined in the following areas.

### Individual patient preferences

There is a growing understanding that different types of cancer require different treatment approaches, especially for metastatic disease. In addition to being incurable, MBC brings other distinct challenges compared with early-stage BC, such as the need for continuous treatment and monitoring; this places a considerable emotional burden on patients as well as a physical burden due to associated side effects and frequent assessments [13].

It is important to recognise that each patient with MBC has individual preferences and needs, whether clinical, social and/or financial. These should be considered routinely when assessing the overall benefit of new MBC treatments and during both clinical and policy decision making, using appropriate instruments [5]. Patients with MBC are the best informants of their individual preferences and needs; identification and consideration of these aspects allows for better patient engagement in the care process, thereby increasing adherence to treatment and minimising disease and psychosocial burden.

The side effects associated with various MBC therapies have led to exploration of the role of patient preferences in decision making with respect to treatment goals and desired outcomes. For many patients, extended survival comes at the expense of diminished health-related quality of life (HRQoL). While aggressive cancer treatment may lead to some clinical benefit, it may also produce significant and burdensome side effects that have a negative impact on HRQoL and the ability to participate in daily life activities [5,14]. Most patients accept an increase in toxicity if the treatment regimen produces a significant survival (OS) benefit, although patients with MBC may interpret the extent of the survival benefit differently to healthcare professionals.

### Quality of life

The overall benefit of any MBC treatment can be conceptualised as the improvement or maintenance of HRQoL combined with robust evidence of efficacy or effectiveness [15]. HRQoL and other patient-reported outcomes (PROs) are useful in differentiating between treatments with similar efficacy or toxicity profiles. These outcomes allow patient-perceived effects to be considered in tandem with clinical efficacy and could provide key differentiation in cases where therapies provide equal survival or other clinical endpoints [16]. Patients’ judgement and assessment of the utility and effects of a treatment are highly valuable for both clinical and policy decision making.

In Canada, the pan-Canadian Oncology Drug Review (pCODR) recognises HRQoL as a highly relevant endpoint and is transparent in its consideration of HRQoL data when assessing the benefit of new oncology treatments [17,18]. In addition, the German Federal Joint Committee expects HRQoL to be included in the benefit assessment dossier [19]. However, these countries are the exception, not the rule. Persuading decision makers to consider HRQoL improvement and thereby pay greater attention to patients’ needs in their decision making process remains challenging because of the absence of effective measurement tools in the MBC setting. Most available HRQoL instruments have been developed for early BC rather than MBC, whereas the tools available for measuring HRQoL in MBC are often not used in clinical trials. The European Organisation for Research and Treatment of Cancer, the European School of Oncology (ESO) and ABC Global Alliance are currently collaborating to develop an MBC-specific HRQoL tool. Promoting the development and use of MBC-specific HRQoL instruments is a critical step in recognising the patient perspective and assessment of patient utility in the decision making process [5].

### Value of delayed chemotherapy

In MBC patients with hormone-receptor-positive and human epidermal growth factor receptor 2-negative disease, there is significant value in delaying the initiation of chemotherapy due to the associated side effects and their negative impact on HRQoL [20,21]. Decision makers in England and Germany have acknowledged the need for new MBC treatments that are effective and have lower toxicity than chemotherapy and that can thus be used in place of chemotherapy [22,23]. For example, one MBC treatment appraisal by NICE in England, took into account patient input: “that people value delaying progression of the disease and an important consideration is delaying the time to chemotherapy” [23]. From the patient’s perspective, delaying chemotherapy, particularly intravenous chemotherapy, is a crucially relevant aspect of the overall benefit of MBC treatment [20].

There is scarcity of literature describing the benefit of delayed chemotherapy as an endpoint. Nevertheless, this SC agreed that delaying the start of chemotherapy in patients with MBC may translate into positive outcomes, such as improvement in HRQoL or a reduction in side effects, depending on the safety profile of alternative treatments [5]. Such a delay may also translate into cost savings for the health system arising from a reduced need for costly emergency department and hospital visits [24,25]. Clinical and policy decision makers should consistently consider delaying both disease progression and the initiation of chemotherapy, thereby reducing exposure to chemotherapy-associated toxicities and side effects among patients in whom certain targeted therapies are indicated. However, not all targeted therapies or patients are alike, and evaluation of their side effects as well as direct comparisons with chemotherapy in terms of efficacy, tolerability and impact on HRQoL are crucial to decision making.

### Patient heterogeneity

Patient heterogeneity, defined as a natural variation between patients that can be attributed to their characteristics, is incorporated into health economic guidelines in European countries [26]. The clinical characteristics of patients with MBC, such as disease severity and comorbidities, should be further incorporated into the overall benefit assessment of MBC treatments to account for differences across sub-populations and in the disease trajectory. The emergence of personalised medicine can be expected to lead to an improved understanding of patient and disease heterogeneity, allowing MBC treatments to be targeted to the patients most likely to benefit from them.

### Side effects and toxicities

Treatment-related side effects and toxicities are an important consideration when assessing the overall benefits of new MBC treatments. They place a significant physical and emotional burden on patients and incur costs for both patients and society [27]. The patients’ wish to avoid certain side effects may be the determining factor when choosing between treatments with similar efficacy. Decision makers should pay greater attention to the patients’ need and the value of different treatment toxicity profiles in relation to the benefits achieved.

### Treatment adherence

Patient adherence to treatment is a key aspect of MBC care and should be further incorporated into the overall MBC treatment benefit assessment. Numerous factors contribute to patients’ adherence to treatment and their treatment administration preferences (i.e. oral vs intravenous) such as convenience or perception of efficacy [28]. Other factors affecting adherence include convenience of administration, costs, patient perception of efficacy, associated side effects (including impact on work and carer duties), patient beliefs, values and past experience [27,28].

### Real-world evidence

Real-world evidence (RWE) has the potential to provide decision makers with additional evidence for the overall benefit of an MBC treatment, allowing more informed decisions when resources are scarce. RWE can contribute towards closing of the efficacy–effectiveness gap by capturing the value of a treatment in clinical practice [29]. Nordon et al. [30] highlighted the importance of identifying real-life contextual patient-, provider- or healthcare-related factors that could impact on effect estimates for medications. RWE could improve patients’ access by reducing payer uncertainty around decisions to adopt a new treatment. Moreover, increased certainty of beneficial outcomes avoids wastage of healthcare resources, which provides healthcare opportunities for other patients [31].

Despite interest from decision makers, the uptake of RWE can be hindered by several barriers, including a lack of clear standards in study design and of infrastructure to collect patient-level data in MBC [31]. The majority of cancer registries only capture aggregate-level data, such as data on diagnosis and death rather than relapse data, meaning it is impossible to ascertain the number of patients with advanced cancer. Few recent cases of regulatory decision making based only on RWE raised concerns about the validity, reliability and the quality of efficacy and safety data submitted. Rapheal et al. (2020) acknowledge that while RWE could speed up the approval process, it could also increase the uncertainty around a treatment’s real benefit to patients [32]. As valuable as RWE is, these barriers limit the potential of RWE to inform decisions and reduce uncertainty around the adoption of new treatments for patients with MBC.

Post-approval RWE can support policy decision makers in providing data on areas of uncertainty such as the burden of illness, natural history of the disease, and the needs of patients in real life versus the trial population (especially older patients who are generally underrepresented in clinical trials). It can also provide useful data on the effectiveness of comparator treatments and how trial surrogate endpoints link with outcomes measured in real life [31]. Evidence from the use of new treatments in clinical practice can go far towards alleviating any uncertainty that policy decision makers may have about the overall benefit of these therapies, and thereby pay greater attention to the patients’ need in a benefit assessment.

### Other considerations in overall MBC treatment benefit assessment

Members of the ABC Global Alliance have called for decision makers to provide better conditions for patients with MBC to return to work [33]. Inclusion of patient experiences, such as their ability to continue working, undertake childcare or other caring duties and maintain autonomy in everyday activities, would increase the value of the overall benefit assessment of MBC treatments. The extent to which decision makers consider patients’ contribution to society, or any other indirect costs of cancer, when assessing a new treatment is unclear. Assessments tend to focus on cost benefits, often measured using direct clinical outcomes only.

In light of these shortcomings, the SC developed a set of recommendations for policy makers, government agencies, HTA decision makers and payers with regard to patient needs in the overall benefit assessment of new MBC treatments. These recommendations are presented in Table 2.

**Table 2:** Key recommendations for government agencies, HTA decision makers, academia and payers to include MBC-specific patient priorities and outcomes in the overall benefit assessment of new MBC treatments.

Key recommendations
• Incorporate the needs of patients with MBC in the overall benefit assessment of an MBC treatment
• Agree on the appropriate endpoints in MBC, including the use of surrogate endpoints such as PFS, with re-evaluation once OS data is available
• Ensure involvement of patients with MBC and, where relevant, voting rights in clinical assessments of MBC treatments at both national and/or regional levels
• Provide means to educate policy decision makers on understanding the needs of patients with MBC
• Incorporate the value placed on delaying the start of chemotherapy in the overall benefit assessment, where applicable
• Support the development of and incorporate MBC-specific HRQoL and MBC-specific PRO measures into decision making and establish one standardised MBC-specific PRO measure that is accepted and used by all HTA agencies
• Support and use observational data collection initiatives in MBC to acquire patient-level data for long-term outcomes
• Recognise and use real-world evidence as supportive evidence in the overall benefit assessment of MBC treatments
• Recognise factors that reduce indirect cancer costs as part of the overall benefit assessment of MBC treatments. For example, the ability of patients with MBC to return to or maintain work or studies, and capacity to participate in daily activities
• Consider objective value framework tools, such as the ESMO Magnitude of Clinical Benefit Scale (MCBS) or the ASCO Value Framework, as one of the sources of information in the decision making process to assess the clinical benefit of new treatments. The methodologies of these tools are constantly being updated according to experience in the field
• Address value in oncology by considering issues such as affordability and value-based pricing and healthcare system adaptability to the rate of innovation in cancer treatment, including waste and the wider healthcare spending

ASCO, American Society of Clinical Oncology; ESMO, European Society for Medical Oncology; HRQoL, Health-Related Quality of Life; HTA, Health Technology Assessment; MBC, Metastatic Breast Cancer; MCBS, Magnitude of Clinical Benefit Scale; OS, Overall Survival; PFS, Progression-Free Survival; PRO, Patient-Reported Outcomes.

### Multi-stakeholder collaboration to improve outcomes for patients

There is also inherent value in multi-stakeholder collaboration in policy decision making. Gannedahl et al. [34] proposed that early and enhanced dialogue with extended stakeholder groups should be a crucial element in supporting the introduction of breakthrough medicines, allowing unmet needs to be addressed and accelerated access to new treatments achieved while maintaining affordability for payers. The European Network for Health Technology Assessment acknowledged that patient engagement and perspective are essential for future collaboration in the benefit assessment of new treatments [35].

Multidisciplinary teams (MDTs) and multidisciplinary tumour boards (MTBs) play an important role in the management of patients with cancer, providing an integrated approach to collaborative cancer care in many countries worldwide [36,37]. The main benefit of MDTs and specialised breast units is that they provide consistent, continuous, coordinated and cost-effective cancer care and give patients with BC access to the best available care and treatments [36,38,39]. Patient-centred care and value-based care are becoming increasingly common in the healthcare arena, and patients should therefore be encouraged to collaborate more with clinicians and other decision makers.

It is crucial that the perspectives and values of patients with MBC are included in the clinical decision making process, the development of treatment guidelines and value-based frameworks. A step towards engaging patients in the decision making process is already evident in the development of the current European School of Oncology and European Society for Medical Oncology (ESO-ESMO) advanced breast cancer guidelines and in the revision process of the ESMO Magnitude of Clinical Benefit Scale (MCBS). ESMO-MCBS is a dynamic tool developed to classify new therapies based on their impact with respect to efficacy, toxicity and HRQoL and its criteria is revised on a regular basis through consultation with various stakeholders, including patients [40].

To further include the patients’ perspective in the decision making, the SC suggested that the development and availability of e-health tools could help determine the added benefit of an MBC treatment. For example, home telemonitoring and personal electronic care plans could improve patient’s participation in the decision making process and provide decision makers with evidence concerning patients’ experiences. Enhanced collaboration between MBC stakeholder groups, namely patients, patient advocacy groups, physicians, researchers, pharmaceutical companies, policy makers and reimbursement bodies, would subsequently improve patient outcomes.

Key recommendations to strengthen multi-stakeholder collaboration including more patient participation to improve MBC patient outcomes are presented in Table 3.

**Table 3:** Key recommendations for MBC multi-stakeholder groups to enhance multi-stakeholder collaboration in order to improve outcomes for patients with MBC.

Key recommendations
• Enforce the importance of specialist Multidisciplinary Teams (MDTs) and Multidisciplinary Tumour Boards (MTBs) in MBC care
• Make sure all patients with MBC are discussed in these boards
• Ensure the patient perspective is integrated into treatment guidelines and enforce the implementation of high-quality, international and national MBC management guidelines
• Further promote participation of patients with MBC in formal early dialogues, integrated scientific advice engagements and (joint) clinical assessments in the EU and beyond
• Provide means and support initiatives to educate patients on understanding the general HTA processes

EU, European Union; HTA, Health Technology Assessment; MBC, Metastatic Breast Cancer; MDTs, Multidisciplinary Teams; MTBs, Multidisciplinary Tumour Boards.

## Discussion

This paper found that not all issues and needs relevant to patients with MBC are consistently pondered in current benefit assessments of new treatments. During expert discussions, the key issues highlighted as being inconsistently considered were the burden of disease, patient preference for a particular treatment, value in the delay of chemotherapy (especially intravenous chemotherapy), drug toxicities, MBC-specific HRQoL, priorities of patients with MBC and supportive evidence for the benefit of treatments from RWE. In addition, discussions highlighted the inherent value of multi-stakeholder engagement between patients, physicians, pharmaceutical companies and regulatory and HTA bodies. Such collaboration could support timely patient access to transformative medicines and potentially improve patient outcomes.

Assessments of the overall benefit of MBC treatments are more valuable when, in addition to factoring in the survival benefit, they are informed by patients’ needs and priorities with respect to HRQoL and ability to participate in daily life activities. However, information about how patients with MBC view the relative importance of improved survival versus greater treatment toxicity is scarce [13]. Previous studies in lung cancer and renal cell carcinoma have suggested that some patients are willing to accept greater toxicity for modest improvements in survival or to live long enough to see a milestone event in their lives, whereas other patients do not consider increased toxicity as acceptable [41,42]. Our expert discussions underscored that preferences of patients with MBC vary with respect to the balance between treatment efficacy and toxicity.

Several HTA agencies, including the Canadian Agency for Drugs and Technologies in Health (CADTH), the UK National Institute for Care and Excellence, the Australian Pharmaceutical Benefits Advisory Committee and the Scottish Medicines Consortium (SMC), currently consider input from patient representatives and patient organisations on patient experience of a disease or health technology, however the weight this input carries in the approval processes is unclear [43]. Although formal training on the HTA process is limited, some agencies (e.g. CADTH and SMC) provide support for patient representatives participating in committees and the writing of dossier submissions however, this type of support is not extended to patient organisations [43]. Thus, there is room for policy decision-makers to become more inclusive and supportive and to provide feedback to patients and patient organisations on the extent of their participation in decision making.

Collaboration between the different MBC stakeholder groups would allow for increased consideration of the priorities of patients with MBC and for improved clinical trial designs and endpoints. Additionally, it would support the development of MBC-specific PROs and facilitate early alignment of requirements for overall treatment benefit assessment in MBC to ensure that the needs of all key decision makers, including patients, are met. Although steps are being taken to improve the overall benefit assessment of new treatments, it is important to promote multi-stakeholder collaboration in both clinical and policy decision making; this will improve outcomes for patients with MBC and patient accessibility to high-quality cancer care.

That said, there are limitations to this paper. The targeted review performed was not protocol-based or systematic, and could have led to selection bias. Moreover, the composition of the SC, with lack of extensive HTA experience and the inclusion of only one biopharmaceutical industry representative, could also have been a source of bias in the discussion of the issues addressed in this paper. It should be noted that the composition of the SC was based on individual willingness to participate in this multi-stakeholder collaboration, and this was thus a self-selecting group.

## Conclusion

Assessments of the overall benefit of MBC treatments are most valuable when informed by patient input on MBC specifics and patient needs and priorities. We call on MBC decision-makers to pay greater attention to patient needs and patient-relevant outcomes, to align on specific patient-relevant evidence requirements in MBC as well as on a common definition of overall treatment benefit. The alignment of, and multi-stakeholder engagement between, patients, physicians, pharmaceutical companies and regulatory and HTA bodies would benefit patients, healthcare systems and society in general.

## Declaration of interests statement

Fatima Cardoso has acted as a consultant on advisory boards for Amgen, Astellas/Medivation, AstraZeneca, Celgene, Daiichi Sankyo, Eisai, GE Oncology, Genentech, GlaxoSmithKline, MacroGenics, Medscape, Merck, Sharp & Dohme, Merus BV, Mylan, Mundipharma, Novartis, Pfizer, Pierre Fabre, prIME Oncology, Roche, Samsung Bioepis, Sanofi, Seattle Genetics and Teva. Laura Biganzoli has acted as a consultant for AstraZeneca, Celgene, Eisai, Genomic Health, Ipsen, Eli Lilly and Company, Novartis, Pfizer, Pierre Fabre and Roche. Kaisa Miikkulainen is employed by ICON plc and Susanne Schuurman was an ICON employed at the time of article submission, and their support was funded by Eli Lilly and Company. Sonia Ujupan is an employee of Eli Lilly and Company. Jenn Gordon has no interests to declare; however, the Canadian Breast Cancer Network has received funding from Amgen, AstraZeneca, Janssen, Merck & Co., Inc., Novartis, Roche and Teva. Nils Wilking has received fees from Jansen, Merck, Sharp & Dome, Novartis and Oasmia for participation in advisory boards and educational activities outside the submitted work. Jaime Espin, Renato Bernardini, Danielle Spence, Sabina Spitz, Nicole Zernik, Sue Chambers and David Peters stated they had no interests in relation to this article, which might be perceived as posing a conflict or bias.

## Role of the funding source

Eli Lilly and Company sponsored the literature review and provided financial support for meeting costs and materials produced by the Steering Committee but did not provide any fees to any of the members of the group for their involvement in this project. Although Eli Lilly and Company has provided comments on this document, the content of the final document reflects consensus from members of the Committee, who have full editorial control.

## Figures and Tables

**Table:** 

Country/region	Website	Website/document link
Europe	Advanced Breast Cancer (ABC) Global Alliance/European School of Oncology (ESO)	https://www.thebreastonline.com/article/S0960-9776(16)30183-7/fulltext
	Association of European Cancer Leagues (ECL)	https://www.europeancancerleagues.org
	“Breast Cancer Matters” website	https://breastcancer-matters.eu
	Council of European Union	https://ec.europa.eu
	European Breast Cancer Network (EBCN)	http://www.ebcn.org
	European Medicines Agency	https://www.ema.europa.eu
	European Union (EU)	https://cancercontrol.eu
	European Network for Health Technology Assessment (EUnetHTA)	https://www.eunethta.eu
	European Reference Organisation for Quality Assured Breast Screening and Diagnostic Services (EUREF)	https://www.euref.org
	European Breast Cancer Coalition (EUROPA DONNA)	https://www.europadonna.org
	European CanCer Organisation (ECCO)	https://www.ecco-org.eu
	European Commission	https://ec.europa.eu
	European Parliament	http://www.ecpc.org
	European Society of Breast Cancer Specialists (EUSOMA)	https://www.eusoma.org
	European Society for Medical Oncology (ESMO)	https://www.esmo.org
	The Value Added Medicines Group, a sector group of Medicines for Europe	http://www.medicinesforeurope.com
Australia	Australasian Society of Breast Physicians (ASBP)	http://www.breastphysicians.org
	Breast Cancer Network Australia (BCNA)	https://www.bcna.org.au
	Cancer Council Australia	https://www.cancer.org.au
	Cancer Drug Alliance (CDA)	www.cancerdrugsalliance.org.au
	Community Affairs References Committee	https://www.aph.gov.au
	Department of Health	http://www.health.gov.au
	Department of Health and Aging	http://www.health.gov.au
	Deloitte	https://medicinesaustralia.com.au https://www2.deloitte.com/au
	F. Hoffmann-La Roche Ltd. Australia	https://www.roche-australia.com
	McGrath Foundation	http://www.mcgrathfoundation.com.au
	National Breast Cancer Foundation	https://nbcf.org.au
	Pharmaceutical Benefits Advisory Committee (PBAC)	https://pbac.pbs.gov.au
	Pharmaceutical Guild of Australia	https://www.guild.org.au
	Tufts University (USA)	https://www.tufts.edu
	University of Sydney	https://sydney.edu.au
Austria	Arbeitsgemeinschaft medikamentöse Tumortherapie (AGMT; (working group on drug tumour therapy)	http://www.agmt.at
	Austrian Breast & Colorectal Cancer Study Group	https://www.abcsg.org
	Austrian Cancer Aid (Österreichische Krebshilfe)	https://www.krebshilfe.net
	Austrian Society for Haematology and Medical Oncology	http://www.oegho.at
	Comprehensive Cancer Center (CCC) Vienna	https://www.ccc.ac.at
	Department of Internal Medicine, University Hospital, Innsbruck, Austria	https://www.i-med.ac.at
	Der Standard	https://www.derstandard.at
	Europa Donna Austria	https://www.europadonna.at
	Federal Ministry of Labour, Social Affairs, Health and Consumer Protection	https://www.bmgf.gv.at
	German Institute of Medical Documentation and Information (DIMDI)	https://www.dimdi.de
	IQVIA	https://www.iqvia.com
	Krebs im Fokus	http://www.krebsimfokus.at
	Ludwig Boltzmann Institute for Health Technology Assessment	http://www.inahta.org
	Medical University of Vienna	https://www.meduniwien.ac.at
Brazil	Associação Brasileira de Portadores de Câncer (AMUCC)	http://www.amucc.org.br
	Brazilian National Cancer Institute (INCA in Portuguese)	https://www.inca.gov.br
	Fundação Oncocentro de São Paulo (FOSP)	http://www.fosp.saude.sp.gov.br
	Institute of Health Technology Assessment (IATS/CNPq), Hospital de Clinicas de Porto Alegre and Graduate Studies in Epidemiology, Federal University of Rio Grande do Sul	http://inct.cnpq.br/web/inct-iats
	Instituto da Mama (IMAMA)	http://institutodamama.org.br
	Instituto do Câncer do Estado de São Paulo (ICESP)	http://www.icesp.org.br
	Instituto Oncoguia	http://www.oncoguia.org.br
	Instituto Se Toque	https://www.atados.com.br
	National Committee for Incorporation of Technologies (Comissão Nacional de Incorporação de Tecnologias; CONITEC)	conitec.gov.br
	Rede Feminina de Combate ao Câncer	https://redefemininasbo.org.br
	Sociedade Brasileira de Cancerologia	http://www.sbcancer.org.br
	Unified Health System (Sistema Unico de Saude; SUS)	http://www.saude.gov.br/sistema-unico-de-saude
Canada	Alberta Health Evidence Reviews	https://open.alberta.ca
	British Columbia Health Technology Review	https://www2.gov.bc.ca/gov/content/health/about-bc-s-health-care-system/partners/health-authorities/bc-health-technology-review
	Canadian Association of Provincial Cancer Agencies (CAPCA)	http://www.capca.ca
	Canadian Breast Cancer Network (CBCN)	https://www.cbcn.ca
	Canadian Cancer Society	http://www.cancer.ca
	Canadian Partnership Against Cancer	https://www.partnershipagainstcancer.ca
	Health Quality Ontario (HQO)	http://www.hqontario.ca
	Institut National d’Excellence en Santé et en Services sociaux (INESSS)	http://www.inesss.qc.ca
	Metastatic Breast Cancer Advocacy in Canada (MBCAC)	http://www.mbcac.ca
	Pan-Canadian Oncology Drug Review (pCODR)	https://cadth.ca
	Quebec Breast Cancer Foundation	https://rubanrose.org
	Rethink Breast Cancer (Rethink)	https://rethinkbreastcancer.com
	Saskatchewan Breast Cancer Connect (SBCC)	http://www.saskbreastcancerconnect.org
France	Department of Medical Oncology, Institut Curie, Paris	https://institut-curie.org
	European Breast Cancer Coalition/Europa Donna Forum France	https://www.europadonna.fr
	French Federation of Comprehensive Cancer Centres (Unicancer)	http://www.unicancer.fr
	French League Against Cancer (Ligue Nationale Contre le Cancer)	https://www.ligue-cancer.net
	French National Cancer Institute (INCa)	https://en.e-cancer.fr
	University Paris VII	https://lillypad.eu/entry.php?e=3336
	Vivre Comme Avant (Breast Cancer Association)	https://www.vivrecommeavant.fr
Germany	BRCA Network	https://www.brca-netzwerk.de
	Breast Cancer Germany (Brustkrebs Deutschland e.V.)	https://brustkrebsdeutschland.de
	Federal Institute for Drugs and Medical Devices (BfArM)	https://www.bfarm.de
	Federal Joint Committee (GBA)	https://www.g-ba.de
	Federal Ministry of Health	https://www.bundesgesundheitsministerium.de
	German Cancer Research Center (Deutsches Krebsforschungszentrum)	https://www.dkfz.de
	Institute for Health Services Research and Health Economics, DDZ, Heinrich Heine University Düsseldorf	https://ddz.de
	Institute for Quality and Efficiency in Health Care (IQWiG)	https://www.iqwig.de
	Mamazone	https://www.mamazone.de
	Mammographie Screening Programm	https://www.mammo-programm.de
	Network of Men with Breast Cancer (Netzwerk Männer mit Brustkrebs e.V.)	https://www.brustkrebs-beim-mann.de
	Paul Ehrlich Institute	https://www.pei.de
	Pink Ribbon Germany (Pink Ribbon Deutschland)	https://www.pinkribbon-deutschland.de
	Rexrodt von Fircks Stiftung	https://www.rvfs.de
	Women selfhelp after cancer (Frauenselbsthilfe nach Krebs)	https://www.frauenselbsthilfe.de
Italy	Aziende Ospedaliere and Private hospitals	https://www.accenture.com
	Coordinamenteo Regionale Unico sul Farmaco (CRUF) of Veneto (Veneto region, EUnetHTA)	https://www.regione.veneto.it/
	Emilia Romagna (Saluter) - Direzione generale sanità e politiche sociali e per l’integrazione	http://www.regione.emilia-romagna.it
	Italian Agency for Pharmaceutical Products (AIFA)	http://www.agenziafarmaco.gov.it
	Italian Oncology Association	http://www.aiom.it
	Local hospitals/Presidi Ospedalieri	Numerous
	Ministry of Economy and Finance	http://www.mef.gov.it
	Ministry of Health	http://www.salute.gov.it
	National Agency for Regional Health Services HTA evaluations (AGENAS)	http://www.agenas.it
	Osservatorio regionale per l’innovazione (ORI)	http://vortal.htai.org
	Pricing and Reimbursement Committee (CPR, under AIFA)	http://www.agenziafarmaco.gov.it
	Piemonte Region HTA organisation	https://www.ires.piemonte.it
	Technical Scientific Commission (CTS, under AIFA)	http://www.agenziafarmaco.gov.it
	Tumore al Seno Metastatico, Noi ci siamo (Association Metastatic Breast Cancer, We are here)	https://www.mbcitalia.com
Mexico	Asociacion Mexicana contra el Cancer de Mama AC “Fundación Cima” (CIMAB)	https://www.cimafundacion.org
	Asociacion Mexicana de Lucha contra el Cancer (Mexican Association to Fight Against Cancer; AMLCC)	http://www.amlcc.org
	Centro Nacional de Excelencia Tecnológica en Salud (CENETEC)	https://www.gob.mx/salud/cenetec
	Consejo de Salubridad General (General Health Council, CSG)	http://www.csg.gob.mx
	Centro Medico Nacional 20 de Noviembre (20th of November National Medical Center)	http://issste-cmn20n.gob.mx
	Federal Commission for Protection against Sanitary Risk (COFEPRIS)	https://www.gob.mx
	Fundacion Salvati	http://salvati.org.mx
	Grupo de Recuperacion Reto	https://www.gruporeto.org
	Latin American and Caribbean Society of Medical Oncology (SLACOM)	http://english.slacom.org
	Latin American Union against Women’s Cancers (ULACCAM)	http://www.ulaccam.org
	National Cancer Institute – INCAN/Instituto Nacional de Cancerologia	http://incan-mexico.org
	National Commission for Social Protection in Health (CNPSS)	http://www.cndh.org.mx
	Secretary of Health (Salud)	https://www.gob.mx
	Tomatelo a Pecho	http://www.tomateloapecho.org.mx
Spain	Agencia Española de Medicamentos y Productos Sanitarios (AEMPS; Spanish agency of medicines and health products)	https://www.aemps.gob.es
	Agència d’Avaluació de Tecnologia i Recerca Mèdiques de Catalunya (AQuAS)	http://aquas.gencat.cat/ca/inici
	Agencia de Evaluación de Tecnologías Sanitarias de Andalucía (AETSA)	https://www.aetsa.org
	Association Against Breast Cancer (AECC)	https://www.aecc.es
	Breast Cancer Foundation (ESMO, reviewed the Spanish guidelines)	https://www.esmo.org/for-patients/patient-guides/breast-cancer
	Federation of Spanish Oncology Societies (FESEO)	http://www.feseo.com
	Fundacion INCLIVA	https://www.incliva.es
	Galician Health Technology Assessment Agency	http://www.inahta.org
	Health Technology Assessment Agency Basque country, Osakidetza	https://www.euskadi.eus
	Ministry of Health, Social Services and Equality (MSSSI)	http://www.msssi.gob.es
	RTI Health Solutions	https://www.rtihs.org
	SOLTI Group	http://www.gruposolti.org
	Spanish Breast Cancer Federation (FECMA)	http://fecma.vinagrero.es
	Spanish Group for Breast Cancer Research (GEICAM)	https://www.geicam.org
	Spanish hospital collaboration	https://www.repositoriosalud.es
	Spanish Society of Gynaecology and Obstetrics (SEGO)	http://www.semnim.es
	Spanish Society of Medical Oncology (SEOM)	https://www.seom.org
	Spanish Society of Senology and Mammary Pathology (SESPM)	http://new.sespm.es
	Unidad de Evaluacion de Tecnologias Sanitarias (UETS)	http://www.comunidad.madrid/servicios/salud/unidad-evaluacion-tecnologias-sanitarias-uets
Sweden	Bröstcancer Förbundet/Bröstcancerföreningarnas Riksorganisation (BRO)	http://www.mynewsdesk.com/se
	Dental and Pharmaceutical Benefits Agency (TLV)	https://www.tlv.se
	Karolinska Institute: Cancer Center Karolinska, Karolinska University Hospital, and Stockholm School of Economics	https://ki.se
	Regionala cancercentrum	https://cancercentrum.se
	Regeringen (Swedish government) - Socialdepartementet	https://www.regeringen.se
	Swedish Agency for Health and Care Services Analysis (Myndigheten för Vård och Omsorgsanalys)	https://www.vardanalys.se
	Swedish Association of Breast Oncologists (Svensk Bröst Onkologisk Förening; BOF)	http://onkologi.org
	Swedish Association of Local Authorities and Regions (SKL)	https://skl.se
	Swedish Breast Cancer Group (Svenska Bröstcancergruppen)	http://www.swebcg.se
S. Korea	Center for New Health Technology Assessment	https://nhta.neca.re.kr
	Health Insurance Review and Assessment Service (HIRA)	https://www.hira.or.kr
	Health and Welfare Committee, National Assembly	http://health.assembly.go.kr
	Korean Association for Health Technology Assessment	http://www.kahta.or.kr
	Korean Breast Cancer Society	http://www.kbcs.or.kr
	Korean Industrial Health Association	https://kiha21.or.kr
	Korean Orphan & Essential Drug Center (KODC)	http://www.kodc.or.kr
	Korean Society for Preventive Medicine	http://www.prevmed.or.kr
	Ministry of Health and Welfare (MOHW)	http://www.mohw.go.kr
	National Cancer Center	https://www.uicc.org
	National Evidence-based Healthcare Collaborating Agency (NECA)	https://www.neca.re.kr
	National Institute of Environmental Research	http://www.nier.go.kr
	Specialised Assessment Committees by Area	https://nhta.neca.re.kr
Taiwan	Chung-Lin Yang (Taiwan, under ISPOR)	https://www.ispor.org/
	Health Promotion Administration	https://www.hpa.gov.tw
	HOPE Foundation for Cancer Care	https://www.ecancer.org.tw
	Ministry of Health and Welfare	http://grb-topics.stpi.narl.org.tw
	Taiwan Society for Pharmacoeconomics and Outcomes Research (TaSPOR)	https://www.taspor.org.tw
UK	Breast Cancer Care UK	https://www.breastcancercare.org.uk
	Breast Cancer Now	https://breastcancernow.org
	Center for Health Economics, University of York	https://www.york.ac.uk/che/
	Department of Health	https://www.gov.uk
	London School of Economics (LSE)	http://www.lse.ac.uk
	Medicines and Healthcare products Regulatory Agency (MHRA)	https://www.gov.uk
	National Institute for Health and Care Excellence (NICE)	https://www.nice.org.uk
	NHS England	https://www.thh.nhs.uk
	UK All Party Parliamentary Group on Breast Cancer	https://www.europadonna.org/public-affairs/national-all-party-groups-on-breast-cancer/united-kingdom/
	UK Breast Cancer Group (UKBCG)	https://ukbcg.org
